# Natural Phytochemicals as Inhibitors of HIF-1α in Breast Cancer: Review of Preclinical Evidence and Future Prospects

**DOI:** 10.3390/cimb48010121

**Published:** 2026-01-22

**Authors:** Ivan Dam, Eric Liu, Abida Ali, Chikezie O. Madu, Yi Lu

**Affiliations:** 1 Department of Biology, University of Memphis, Memphis, TN 38152, USA; ivandamwsms@gmail.com (I.D.); yammytheduck@gmail.com (E.L.); alikhanabida@gmail.com (A.A.); 2 Department of Biological Sciences, University of Memphis, Memphis, TN 38152, USA; comadu@memphis.edu; 3 Department of Pathology and Laboratory Medicine, University of Tennessee Health Science Center, Memphis, TN 38163, USA

**Keywords:** phytochemicals, HIF-1α, polyphenols, alkaloids, isothiocyanates, natural product, angiogenesis, breast cancer

## Abstract

Breast cancer is the most prevalent form of cancer among women globally. The hypoxic microenvironment resulting from the rapid oxygen consumption of rapidly dividing cancer cells causes the accumulation of hypoxia-inducible factor-1α (HIF-1α) due to reduced catalytic activity of prolyl hydroxylase domain 2 (PHD2) and Von Hippel-Lindau (VHL). Under physiological conditions, HIF-1α regulates cell response to hypoxic environments. Activating genes are involved in glycolysis, angiogenesis, and erythropoiesis. However, the sustained hypoxic environment in breast cancer facilitates metastasis, immune evasion, and drug resistance. Consequently, HIF-1α is a key target in breast cancer treatment, and such inhibitors of HIF-1α may prove to be a viable treatment option. Increasing evidence suggests that natural chemicals, such as polyphenols, isothiocyanates, curcumin, and alkaloids, are inhibitors of HIF-1α. Preclinical studies using animal models and breast cancer cell lines indicate significant reductions in angiogenesis, despite challenges of heterogeneity, bioavailability, and dose optimization. This review intends to summarize current evidence on natural inhibitors of HIF-1α and potential future studies.

## 1. Introduction

With 2.3 million cases reported in 2022, breast cancer is a major cause of cancer-related mortality among women. The disease is not specific to underdeveloped countries. Instead, the incidence increases with higher Human Development Index (HDI) values. The mortality rates, however, are disproportionately higher in countries with lower HDI due to disparities in early diagnosis and access to advanced treatment methods [1]. Additionally, tumor metastasis is a major contributor to breast cancer mortality. Autopsies have revealed that almost seventy percent of breast cancer patients discover a metastasized tumor in the bones or lungs [2]. Because metastasis is critical to tumor mortality, early targeting of the tumor in its progression can limit its growth and spread and significantly reduce mortality rates.

Tumor growth and metastasis are dependent on a sufficient supply of oxygen and nutrients through blood vessels. The uneven distribution of oxygen is common in tumors. The tumor cells located close to blood vessels receive more than sufficient oxygen to survive, whereas cells farther away experience necrosis due to hypoxia [3]. These hypoxic regions enhance tumor progression through neovascularization. Angiogenesis, the formation of new blood vessels, is crucial to tumor growth and metastasis. Tumors require new vasculature, which supplies oxygen and nutrients, to expand beyond the 1–2 mm [4].

Hypoxia is an important characteristic of tumors due to the tortuous and insufficient blood vessels. Under normoxic conditions, HIF-1 cannot form, despite the continuous expression of HIF-1β. This is because the HIF-1α gets hydroxylated by PHD2. VHL binds to the hydroxylated HIF-1α and recruits an E3 ubiquitin protein ligase to mark HIF-1α for destruction through polyubiquitination. However, in a hypoxic tumor microenvironment, HIF-1α degradation slows down due to decreased hydroxylation by PHD2 in the absence or lack of oxygen [3]. This results in an accumulation of HIF-1α, which binds with the continually produced HIF-1β to form HIF-1. HIF-1 enters the nucleus where it binds to Hypoxia Response Element (HRE) as a transcription factor, upregulating genes that promote proliferation, angiogenesis, migration, and Epithelial–Mesenchymal Transition (EMT) of tumor cells and resistance to radiation therapy [2,3]. EMT results in increased motility of tumor cells due to weakened junctions between neighboring cells. This allows the tumor to migrate over long distances inside the body [5]. Therefore, angiogenesis promotes tumor metastasis through EMT by providing motile tumor cells with blood vessels as a pathway to travel throughout the body.

The production of HIF-1, the master regulator of hypoxia-inducible genes, is regulated by the degradation of HIF-1α [6]. Therefore, HIF-1a is the true master regulator of hypoxia-inducible genes. Targeting HIF-1α will lower tumor vascularization by decreasing VEGF production. Neovascularization is an important step in tumor growth and metastasis, both of which contribute to the high mortality of breast cancer cases. This is primarily because, as the tumor grows larger, the cells need new vasculature to receive nutrients and proliferate as diffusion from neighboring cells becomes ineffective [7]. Without the formation of any new vasculature, the tumor cells will begin to die in a state of hypoxia. The primary pro-angiogenic target of HIF-1 is vascular endothelial cell growth factor (VEGF). This growth factor binds to the VEGF receptor, which is located on the membrane of endothelial cells, thereby promoting their proliferation. This directly promotes tumor neovascularization [6]. Since HIF-1α expression can result in tumors becoming resistant to common treatments such as chemo and radiotherapy, phytochemicals emerge as an attractive method to be explored.

In addition to inhibiting HIF-1α, phytochemicals also suppress carcinogenesis through oxidative stress and epigenetic regulation. Carcinogenesis can be induced through oxidative stress, resulting in gene mutations and chromosomal anomalies [8]. Many phytochemicals reduce oxidative stress by acting as monofunctional inducers, selectively stimulating phase II metabolic enzymes [9]. Phase I metabolism produces highly reactive and genotoxic products through oxidation, reduction, and hydrolysis of xenobiotics. Phase II enzymes use the highly reactive products of phase I metabolism as substrates to form less-reactive conjugates [10]. While some phytochemicals act as bifunctional inducers, stimulating both phase I and phase II enzymes, many others are monofunctional inducers that selectively stimulate phase II enzymatic activity, thereby reducing oxidative stress and, consequently, the risk of carcinogenesis [9]. The anti-tumor effects of phytochemicals go beyond managing oxidative stress. A study conducted on breast cancer cells used Pterostilbene. This phytochemical resulted in increased DNA methylation at oncogene enhancers and decreased H3K36me3 (active marker) binding at oncogene enhancers, overall decreasing oncogene expression [11]. Therefore, phytochemicals attack tumors in several different ways, highlighting their potential and encouraging further research.

## 2. Introduction to Phytochemicals

Phytochemicals can be classified into various subgroups.

Polyphenols are plant-derived compounds recognized for their antioxidant properties and potential in cancer treatment [12]. Polyphenols are broadly classified based on their chemical structure into groups such as flavonoids (including catechins and flavonols) and stilbenes, both of which exhibit anti-inflammatory and antioxidant properties relevant to cancer biology [13,14]. In the context of hypoxia, polyphenols play a crucial role by reducing the accumulation of reactive oxygen species (ROS), which otherwise contributes to the stabilization of HIF-1α [15,16]. Among the most well-studied polyphenols with anti-HIF-1α effects are resveratrol, epigallocatechin gallate (EGCG), and quercetin.

The antioxidant activity of phytochemicals allows for the neutralization of ROS and a decrease in tumor progression. Although ROS play an essential role in signalling and immune defense pathways, their accumulation can result in oxidative damage to DNA, intracellular proteins, and lipids [8,17]. Since DNA damage can result in mutations and chromosomal aberrations, which can lead to the formation of a tumor, the accumulation of ROS is a contributing factor in carcinogenesis [18]. Polyphenols reduce oxidative stress by stabilizing ROS and reducing oxidative stress due to their phenolic structure, which consists of multiple hydroxyl groups attached to aromatic nuclei. The resonance in the aromatic ring allows the compound to stay stable after donating a hydrogen to a free radical. In the end, the free radical is neutralized, and the free radical chain reaction is inhibited because of the electron delocalization in the aromatic ring of the polyphenol [19].

## 3. HIF-1α Biology and Mechanistic Pathways of Action

Hypoxia-inducible factor is a heterodimer transcription factor in which one of three oxygen-sensitive α subunits (HIF-1α, HIF-2α, and HIF-3α) [7] and dimerizes with one of two oxygen-insensitive β subunits (HIF-1β and HIF2β), also known as aryl hydrocarbon nuclear translocator subunits [20]. Although all HIF isoforms regulate gene expression under hypoxic conditions, expression and functionality vary significantly. HIF-2β is mainly expressed in select tissues such as neurons [20]. Although HIF-2α shares structural similarities with HIF-1α, it exhibits different functionality across different tumors and cell types. Whereas HIF-1α’s role of regulating glycolysis and genes involved in energy metabolism leads to distinct effects of activation promoting tumor growth and metastasis in tumor cells, and deletion reduces tumor growth, HIF-2α effects in tumor models are more variable; deletion increases tumor progression, while overexpression can also promote angiogenesis [21]. Consequently, much research has focused on HIF-1α, the most extensively expressed subunit of HIF-1 in mammalian cells [22], particularly due to its association with solid tumors [7].

**Figure 1:**
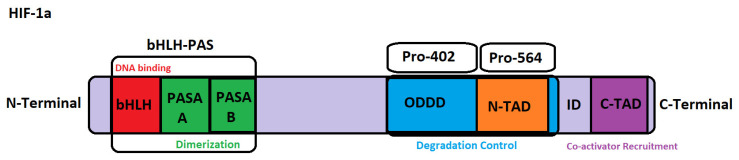
Representation of the domains of Hif-1α.

Structurally, HIF-1α is a 120–130 kD protein made up of 826 amino acid polypeptides. The N-terminal of HIF-1α subunits contains basic helix-loop-helix domains (bHLH), Per-ARNT-Sim-A (PAS-A), and Per-ARNT-Sim-B (PAS-B) domains [23]. The bHLH-PAS (Figure 1) domain mediates the dimerization of α and β subunits as well as binding to hypoxia response elements (HREs) [24]. Sequentially downstream of the bHLH-PAS domain is an oxygen-dependent degradation domain (ODDD) consisting of approximately 200 amino acid residues. Portions of this domain—notably Pro-564 and Pro-402 (Figure 1)—independently confer degradation of HIF-1α under normoxic conditions; consequently, deletion of the ODDD would give rise to a stable HIF-1α capable of dimerization, binding to HREs, even in the absence of hypoxic signaling [25]. Further downstream. HIF-1α has two independent transactivation domains (TAD) enriched with acidic and hydrophobic amino acids and linked by an inhibitory domain, the NH2-terminal N-TAD, which overlaps with the ODDD and has functions under both hypoxia and normoxic conditions, whereas the COOH-terminal TAD (C-TAD) (Figure 1), containing an asparagine residue, only functions under hypoxic conditions [26]. The inhibitory domain sequences situated between prevent transcriptional activation by TAD [27].

Under normoxic conditions, HIF-1α is continuously synthesized but rapidly degraded due to post-translational hydroxylation of Pro-564 and Pro-402 by prolyl hydroxylase domain-containing proteins (PHD1, PHD2, and PHD3) [28]. The hydroxylation of the proline residues provides a binding site for the von Hippel-Lindau protein (pVHL) [29]. The pVHL associates with the E3 ubiquitin ligase complex, which is composed of Elongin B and C, Cullin 2, and RING-box protein-1 [30]. The E3 works in conjunction with E1 ubiquitin-activating enzyme and E2 ubiquitin-conjugating enzyme to attach a chain of active ubiquitin to HIF-1α [31]. Ubiquitin-dependent 26S proteasomes subsequently degrade the polyubiquitinated HIF-1α; however, studies have found that HIF-1α degradation in ischemic neurons is mediated by 20S proteasomes [32]. In addition to the hydroxylation of the proline residue, the asparagine residue 803 of the C-TAD is hydroxylated by factor-inhibiting HIF (FIH), preventing the binding of HIF-1α to co-activators p300 and CBP.

Under hypoxic conditions, due to insufficient molecular oxygen needed as a substrate for the hydroxylation and subsequent degradation of HIF-1α, FIH activity is also inhibited by the low oxygen. Thus, the stabilized HIF-1α moves into the nucleus, associating with HIF-1β to form a transcriptionally active heterodimer [33]. Asn 803, along with several hydrophobic residues, Ile-802, Leu-808, Leu-814, Leu-815, and Leu-818 found in C-TAD, help bind the coactivator to the CH1 domain of CBP to HIF-1α [27]. p300 also binds to HIF-1α by its Ch1 domain. The transcriptional complex then facilitates the binding of HRE, increasing transcriptional activity of target genes [29].

In addition to oxygen availability, reactive oxygen species (ROS) also influence the hydroxylation of HIF-1α, as elevated ROS levels reduce the availability of Fe^2+^, Ascorbate (ASC), both hydroxylation cofactors of PHD & HIF [34], diminishing ODDD and C-TAD hydroxylation, resulting in stabilized HIF-1α even in nonhypoxic conditions.

Regulation of HIF-1α can also be done upstream and downstream of the protein, as PI3K/Akt and MAPK/ERK are growth-factor-driven pathways that control HIF1-1a translation and expression independently of oxygen levels [35]. VEGF and MMp-2/-0 are canonical targets of HIF-1α [36]. Regulation of HIF-1α abundance can also be regulated upstream through targeting growth-factor-driven synthesis. As the PI3k/Akt/mTORC1 pathway promotes synthesis of HIF-1α protein on both the transcriptional level and translation level [37], and the MAPK/ERK pathway is necessary for the transactivation activity of HIF-1α and the transactivation activity of p300 [38]. In addition to the downstream regulation of HRE, notably vascular endothelial growth factor (VEGF) and metalloproteinase 2 (MMP-2), among other genes involved in angiogenesis, such as cathepsin D and keratin, are targets of the HIF-1α transcriptional complex [38], as HIF-1α levels proportionally increase with these proteins.

The expression of HIF-1α is also varied based on the type of breast cancer; the main subtypes considered for the review are estrogen receptor positive (ER+), human epidermal growth factor 2 positive (HER2+), triple-negative breast cancer (TNBC), and Luminal B-like breast cancer, [39] which is also characterized by the overexpression of ER, but is typically more proliferate and aggressive than Luminal A. Studies found that HER2+ tumors showed the highest levels of expression of HIF-1a, with TNBC expressing moderate amounts, and ER+ and Luminal type having the lowest HIF-1α levels, despite showing no statistically significant difference from TNBC [40]. But despite the differences in expression, the consistent and definite expression of HIF-1α makes it a valuable target across the studied breast cancer subtypes.

## 4. Phytochemicals with Anti-HIF-1α Activity

Resveratrol (RSV) (3, 5, 4′-trihydroxystilbene) is a naturally occurring stilbene polyphenol produced by many plants, such as grapes, apples, blueberries, plums, and peanuts, which has attracted considerable attention for its anticancer activities [41]. For example, resveratrol suppresses cancer cell proliferation, induces apoptosis, and growth arrest, effects typically due to inhibition of the PI3K/Akt signaling pathway [42]. In addition to suppressing the PI3K/Akt pathway, resveratrol has been reported to inhibit MAPK/ERK and p38 MAPK signaling, further contributing to its pro-apoptotic and anti-proliferative effects [43,44]. Importantly, RSV reduces HIF-1α protein accumulation without affecting its mRNA transcription, linking its anticancer effects to the inhibition of hypoxia-driven pathways in tumor cells [45]. By reducing HIF-1α buildup, resveratrol has been shown to inhibit proliferation and induce apoptosis, specifically in breast cancer cell lines MCF-7 and MDA-MB-231 [46,47]. By reducing H1F-1α protein accumulation, the expression of genes such as vascular endothelial growth factor (VEGF) is limited, a key factor that typically promotes angiogenesis in tumors [45]. Additionally, HIF-1α promotes the expression of matrix metalloproteinases such as MMP-2 and MMP-9, enzymes that facilitate tumor growth by degrading the extracellular matrix and enhancing angiogenesis [48,49]. By reducing HIF-1α protein accumulation, resveratrol may limit MMP and VEGF expression, thereby inhibiting tumor growth and angiogenesis, though direct evidence in breast cancer is still emerging. Table 1 below shows the structure of RSV.

EGCG, the predominant catechin in green tea, inhibits HIF-1α stabilization and VEGF expression, which have a critical role in breast cancer tumor angiogenesis [52,53]. By reducing reactive oxygen species (ROS), EGCG may inhibit HIF-1α protein accumulation, thereby facilitating HIF-1α protein degradation via the VHL-mediated ubiquitin-proteasome pathway, although direct evidence in breast cancer cells remains limited [54,55,56]. This reduction in HIF-1α protein levels therefore decreases VEGF expression, which impairs angiogenesis, thus limiting the tumor’s ability to support growth and metastasis [57]. Table 1 below shows the structure of EGCG.

Quercetin is a flavonoid commonly found in apples, dill, berries, cilantro, lovage, and onions, is recognized for “antioxidant, antimicrobial, anti-inflammatory, antiviral, and anticancer properties” [63]. Like other polyphenols previously mentioned, quercetin has been shown to inhibit HIF-1α stabilization under hypoxic conditions, thus reducing downstream pro-angiogenic signaling, including VEGF expression [59]. Quercetin is also known for its ROS scavenging property, which contributes to its ability to modulate oxidative stress-related signaling pathways such as PI3K/Akt and MAPK, both crucial intracellular signaling cascades that regulate cell growth, proliferation, and survival, which are overexpressed in cancer, thereby inhibiting cancer growth, proliferation, and survival [60]. By reducing ROS, quercetin may promote HIF-1α hydroxylation, thereby decreasing HIF-1α stability [61]. Table 1 below indicates the structure of Quercetin.

Curcumin, a polyphenol found in turmeric, is also found to suppress HIF-1α and downstream targets of VEGF, thereby inhibiting angiogenesis caused by hypoxia [65,74]. Although both sulforaphane and curcumin show promise in inhibiting HIF-1α and VEGF, further research must be carried out to support similar findings in breast cancer. Table 1 below shows the structure of curcumin.

Isothiocyanates are another class of compounds derived from glucosinolates in broccoli, cabbage, and cauliflower [75]. These compounds are generated when the vegetables are chewed or chopped, which activates the enzyme myrosinase [75]. Additionally, they have notable anti-HIF-1α activity, like the polyphenols mentioned previously [76]. Sulforaphane, an isothiocyanate derived from cruciferous vegetables, has demonstrated anti-HIF-1α activity, which may be through the Nrf2/Keap1 pathway [68,69]. Table 1 below shows the structure of sulforaphane.

Alkaloids are a class of nitrogen-containing natural products found in plants such as *Berberis* species that have shown inhibitory effects on hypoxia signaling in cancer models. For example, berberine, an isoquinoline alkaloid, has been reported to reduce HIF-1α expression at both the mRNA and protein levels and to suppress hypoxia-associated phenotypes in breast cancer cells [72,73]. Berberine’s anti-HIF-1α effects are accompanied by downregulation of downstream targets such as VEGF and inhibition of angiogenic and survival pathways, suggesting that it interferes with hypoxia-responsive tumor adaptation [77]. Although mechanistic and in vivo breast cancer data remain limited, these findings indicate that alkaloids like berberine represent an additional class of phytochemicals capable of modulating HIF-1α–driven hypoxic responses. Table 1 below shows the structure of berberine.

## 5. Preclinical and Clinical Evidence

**Figure 2:**
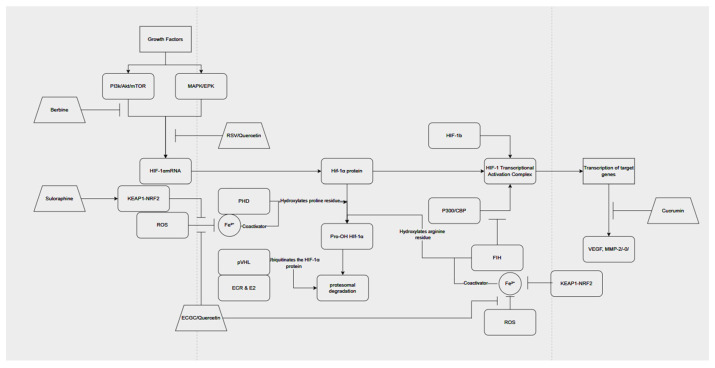
HIF-1α pathway and points of Phytochemical inhibition.

Breast cancer is a heterogeneous disease comprising multiple molecular subtypes, including estrogen receptor–positive (ER+), human epidermal growth factor receptor 2–positive (HER2+), and triple-negative breast cancer (TNBC), each characterized by distinct biological behaviors and therapeutic vulnerabilities [1,39]. HIF-1α signaling plays differential roles across these subtypes, particularly in hypoxia adaptation, angiogenesis, metabolic reprogramming, and treatment resistance [2,3,7,21]. Many in vitro studies discussed in this review utilize ER+ (MCF-7) and TNBC (MDA-MB-231) models, suggesting that phytochemicals such as resveratrol, EGCG, quercetin, curcumin, sulforaphane, and berberine may be especially relevant to these subtypes. Figure 2 shows points of inhibition through these phytochemicals. In contrast, evidence in HER2+ breast cancer remains limited, highlighting an important gap for future investigation.

Resveratrol has been extensively studied in breast cancer cell lines. In vitro, the resveratrol analogue HS-1793 was shown to inhibit hypoxia-induced HIF-1α expression in breast cancer cell lines (MCF-7 and MDA-MB-23) more than normal resveratrol, acting at a post-transcriptional level [50]. In vivo, HS-1793 significantly suppressed the growth of breast cancer xenografts, while also downregulating Ki-67 and VEGF [50]. Clinically, however, evidence remains limited, as studies in humans typically investigate other biomarkers instead of HIF-1α, with no clinical trials directly demonstrating reduced HIF-1α within breast cancer tumors. Additionally, in other clinical trials, Resveratrol’s limited bioavailability has remained a significant obstacle in achieving significant therapeutic outcomes [51].

Green tea catechins, such as EGCG, have also been studied extensively. In vitro, EGCG decreased the expression of HIF-1α and VEGF in MCF-7 breast cancer cells, inhibiting angiogenesis and cell growth [57]. In vivo, animal models inoculated with E0771 breast cancer cell line, a luminal-B like model, further confirms EGFG’s inhibition of angiogenesis and breast cancer progression due to lesser levels of VEGF and HIF-1α, along with NFκB [52]. Clinically however, a pre-surgical trial of green capsule vs. no green capsule in postmenopausal breast cancer patients with ductal carcinoma in situ (DCIS) or early-stage (I/II) invasive breast cancer, indicated a decline in cell proliferation (Ki-67) for women taking green tea supplements, but no significant changes were observed in apoptosis or angiogenesis. These findings suggest that while EGCG shows promising preclinical activity, transitional relevance in humans still remains uncertain, and further clinical studies are needed to directly evaluate HIF-1α modulation [58].

In silico, quercetin has been hypothesized to increase the degradation of HIF-1α protein [62]. At the preclinical level, in vitro, quercetin inhibits hypoxia-related HIF-1α protein accumulation and VEGF release in breast cancer cell lines primarily through inhibition of HIF-1α protein synthesis [63]. In vivo, quercetin significantly suppressed HIF-1α in a hypoxia-dependent manner, but not in normal cells [64]. Clinically however, studies testing this hypothesis are currently lacking, emphasizing a need for future research. These findings indicate promising preclinical activity but emphasize the need for additional validation for both in vivo validation and clinical investigation before translational findings may be established.

Curcumin is one of the most broadly studied phytochemicals. In vitro, its EF24 analogue has been shown to decrease HIF-1α protein and disrupt hypoxia signaling in MDA-MB-231 breast cancer cells [66]. In vivo studies in other cancers have shown that curcumin can down-regulate HIF-1α; however, breast cancer has not yet been studied extensively in this context [65]. Clinically, although evidence in this context is lacking, curcumin, alongside chemotherapy, has been found to reduce the severity of radiation dermatitis in breast cancer patients with noninflammatory invasive disease or carcinoma in situ, without molecular subtype stratification (e.g., ER+, HER2+, TNBC) [67].

In vitro, Sulforaphane has been found to reduce the size and number of primary mammospheres in SUM159 and MCF7 breast cancer cells, which are TNBC and ER+, respectively, while downregulating VEGF and HIF-1α in breast cancer cells [70]. In vivo, sulforaphane had reduced breast cancer tumor size by 50%, still associated with downregulation of VEGF and HIF-1α in breast cancer, and still using both SUM159 and MCF7 breast cancer cells [70]. Clinically, sulforaphane in a placebo-controlled, double-blinded, randomized design of a population of postmenopausal breast cancer patients, most of whom had an early-stage breast cancer (I/II) that was predominantly ER+, and HER2- indicated a decline in Ki-67 (cell proliferation), yet no biomarkers were statistically significant. Additionally, clinical trials testing the effect of sulforaphane on HIF-1α in breast cancer cells are still limited, highlighting the need for future research [71].

In vitro, Berberine has been found to reduce the chemoresistance of breast cancer using the MCF-7 cell line at a concentration greater than 20 µM [73]. Interestingly, berberine had negligible effect at concentrations less than 10 µM. In vivo, hypoxia-induced, drug-resistant MCF-7 breast cancer xenograft models demonstrated that berberine treatment reduced tumor volume and weight [73]. Specifically, low-dose berberine combined with doxorubicin (Ber-L+DOX) and high-dose berberine (Ber-H) significantly decreased tumor growth compared with controls, whereas low-dose berberine alone had minimal effect [73]. In vivo, in xenograft models, low-dose berberine significantly induced the inhibition of AMPK and down-regulated the expression of HIF-1α, but high-dose berberine promoted the expression of p53 by inhibiting AMPK- HIF-1α signaling pathway [73]. Clinically, evidence is lacking, but berberine has been found to reduce the chemoresistance of breast cancer both in vitro and in vivo.

The studies discussed suggest that phytochemicals emerge as a promising therapeutic drug. However, additional clinical evidence is needed to ensure their targeting of HIF-1α in breast cancer. Although most of the phytochemicals mentioned appear to suppress or downregulate HIF-1α, there is a lack of sufficient clinical evidence, which currently restricts their translation into clinical settings.

Among the phytochemicals discussed, EGCG, resveratrol, and sulforaphane exhibit distinct translational trajectories with respect to HIF-1α inhibition in breast cancer. EGCG demonstrates consistent suppression of HIF-1α and downstream angiogenic signaling across in vitro and in vivo models. However, clinical studies to date suggest predominantly antiproliferative rather than anti-angiogenic effects, potentially reflecting limited systemic bioavailability. Resveratrol, particularly through stabilized analogues such as HS-1793, exhibits potent post-transcriptional inhibition of HIF-1α and robust antitumor effects in xenograft models, yet its clinical translation remains constrained by rapid metabolism and poor bioavailability. In contrast, sulforaphane appears to exert broader effects on hypoxia-associated tumor phenotypes, including mammosphere formation and tumor burden reduction, and benefits from comparatively favorable absorption; nonetheless, interindividual variability and inconsistent biomarker modulation in clinical trials limit definitive conclusions regarding its anti–HIF-1α efficacy. Collectively, these differences highlight that while resveratrol analogues may offer the greatest mechanistic specificity, sulforaphane may possess comparatively greater translational feasibility, underscoring the need for direct HIF-1α assessment in future clinical studies.

Overall, while multiple phytochemicals exhibit consistent anti-HIF-1α effects in vitro and in vivo, the absence of well-designed clinical trials directly measuring HIF-1α activity in breast cancer tumors precludes definitive conclusions regarding their therapeutic efficacy. In addition, accurately modeling the environment in which the phytochemicals will act is essential to enhancing the efficacy for clinical translation. Consequently, more studies utilizing organoids and xenograft models are crucial to simulating the complex hypoxic tumor environment. Studies incorporating these models better evaluate the limitations of phytochemicals when administered in vivo, which show promising chemopreventive activity in vitro.

Table 1 provides a summary of chemical formulas, mechanisms of HIF-1α inhibition, in vitro, in vivo, and clinical evidence, along with the breast cancer subtype/model used for each phytochemical discussed.

## 6. Challenges and Future Directions

The antitumor activity of phytochemicals is not restricted to breast cancer. Proanthocyanidins derived from grape seed extracts have demonstrated anti-tumor effects in human colorectal carcinoma, prostate cancer, and squamous cell carcinoma of the head and neck [78]. Similarly, apples are rich in bioactive compounds such as quercetin, catechin, phloridzin, and chlorogenic acid, all of which also exhibit anticancer activity [79]. Epidemiological studies show that daily consumption of apples and pears is associated with a reduced risk of lung cancer in women [80]. In addition, anthocyanins and other polyphenols present in sweet potato leaves also exhibit anticancer effects in several cancer models, including breast, prostate, colorectal, colon, cervical, and lung cancers [81].

Despite the effectiveness of phytochemicals such as curcumin, resveratrol, and EGCG in inhibiting neovascularization by modifying pathways like MAPK and JAK/STAT3, some challenges prevent them from entering mainstream treatment methods. Their multi targeting and low bioavailability are important factors restricting their use in cancer therapies.

Compared to low bioavailability, the multi-targeting of phytochemicals significantly limits their use in targeted therapy. Quercetin exemplifies this issue as it can bind to enzymes with distinct structures, such as phosphatidylinositol 3-kinase, helix-turn-helix-type transcriptional regulator, and 3-hydroxyisobutyryl-CoA hydrolase, due to the lack of diversity in binding site structure [82]. This lowers its efficacy in targeted therapies. When administered at low concentrations, phytochemicals are unlikely to accumulate in sufficient amounts at the tumor site. Higher concentrations distribute the dose across multiple pathways rather than selectively targeting the tumor site. This results in a reduced effective concentration at the target site [83]. In addition, multi-targeting might allow the drug to bind to off-target proteins at the tumor site, further lowering the effectiveness. This makes multi-targeting a major obstacle in translating phytochemicals into targeted cancer therapies.

One of the biggest challenges in achieving therapeutically relevant concentrations of phytochemicals in humans is their low bioavailability. Lipinski’s rule of five is used to predict that high bioavailability is not fulfilled by polyphenols such as curcumin, predicting that they have low bioavailability [84,85]. In addition to the predictions, even when these phytochemicals were tested in vivo, they failed to produce the same effect as they did in vitro studies [64]. More specific examples are listed below.

Resveratrol’s low bioavailability results in insufficient accumulation even after dose escalation to 5000 mg [86]. In vitro studies have demonstrated that a 5 µmol/L dose of resveratrol is enough to induce its chemopreventive effects. In vivo, most (75%) of the dose is absorbed. However, it gets rapidly metabolized into resveratrol glucuronides and sulphates in the intestines and liver during phase II metabolism [87].

In vivo studies involving quercetin demonstrate that it is easily absorbed, with approximately 93% of the orally administered dose being absorbed within one hour in rat models. However, the low bioavailability stems from glucuronidation and sulfation during phase II metabolism when quercetin is rapidly metabolized into glucuronides and sulfoglucuronides [88].

Similarly, a clinical trial involving individuals with high-risk precancerous conditions found that plasma concentrations of curcumin remained low even after doses as high as 8000 mg/day. In addition to the low bioavailability, the high dose of curcumin also resulted in pill burden for the participants in the study [89]. This study highlights that the low bioavailability of administering curcumin orally and its unrealistically high doses impede its clinical translation in cancer therapies.

Berberine’s low bioavailability is also a barrier preventing its clinical translation. Studies conducted on rats report an oral bioavailability of less than 1% [90]. In contrast to resveratrol and quercetin, which are mainly metabolized during phase II metabolism, berberine undergoes rapid first-pass metabolism in the gut and liver. Additionally, it is also quickly eliminated from the body. For instance, it takes 1.13 h to be cleared from the plasma and 12 h to be removed from the hippocampus [90].

Several factors contribute to the low bioavailability of phytochemicals, including low solubility, poor absorption, instability due to variations in gastric and colonic pH, metabolism by gut microflora, active efflux mechanisms, and first-pass metabolism [85,91,92]. Phytochemicals are rapidly conjugated through glucuronidation in the intestine and liver under phase II metabolism [85]. Glucuronidation is a major process in the elimination of substances, including from the body. In this detoxification process, Uridine diphosphate glucuronosyltransferases (UGT) attach glucuronic acid to compounds to turn them into hydrophilic glucuronides, which are then excreted through urine or bile [93]. To prevent elimination through phase II metabolism, combination therapies could act as a solution. Due to phase II metabolism, phytochemicals like resveratrol are rapidly metabolized and have low bioavailability despite having a high absorption rate in the small intestine [94]. Administering phytochemicals with drugs that slow down the metabolism of the phytochemicals by blocking the glucuronidation pathway is likely to result in a higher therapeutic effect [85]. Shoba shows a significant spike in plasma curcumin levels when administered with piperine, which is an inhibitor of the glucuronidation pathway. When administered alone, the curcumin level was near negligible [95].

Despite the promising results, it is important to be cautious of the long-term effects of inhibiting the metabolism of xenobiotics. Detoxification can be inhibited by compounds such as piperine, allowing carcinogens to accumulate in the body and resulting in counterproductive effects [85]. Nanoparticle (NP) encapsulation emerges as a solution to this problem. It is a safer alternative that protects the compound from degradation and enhances drug distribution. Encapsulation in polymeric nanoparticles increases the circulation half-life of the drug through their hydrophilic barrier, which allows them to escape opsonization [96]. For instance, Shaikh administered curcumin through nanoparticles and recorded a nine-times increase in oral bioavailability than administering curcumin with piperine [97].

There are multiple types of NPs, such as micelles, polymeric dendrimers, quantum dots (QDs), microspheres, nanoemulsions, hydrogels, and liposomes [91]. These can actively target the tumor through a ligand-mediated approach, ensuring that the drug is only delivered to cancer cells. NPs identify and target tumor cells through surface proteins specific to these cells [98]. However, targeting a single ligand isn’t enough. A study showed that gold NPs targeting two ligands were more selective at drug delivery than those targeting just one [99]. Other factors must also be kept in mind, such as ligand density’s impact on avidity. Though active targeting is a huge advantage of using NPs for drug delivery, they have to be specifically designed for each phytochemical and even patients based on their tumor type and subtype.

Multiple studies have demonstrated that nanoparticle (NP) encapsulation significantly enhances the potential of phytochemicals like curcumin as a drug. Umerska et al. compared the effect of NPs in a colon cancer cell line and found that Eudragit RLPO (ERL) NPs yielded the best results when compared to polylactic-co-glycolic acid (PLGA) and polycaprolactone (PCL) NPs. Although ERL NPs exhibited significantly lower encapsulation efficiency (62%) than PLGA (90%) and PCL (99%) NPs, they compensated for it through a faster drug release, with almost 57 ± 7% of curcumin being released within 5 min, as well as fully releasing all the encapsulated curcumin [100].

Another investigation carried out by Chang examined Glycyrrhetinic acid-modified cat-ionic liposomes as a drug delivery method both in vitro and in vivo. Curcumin was encapsulated in glycyrrhetinic acid-modified cationic liposomes (GAMCLCL) and administered to liver cancer cell lines and tumor-bearing mice. In vitro, GAMCLCL exhibited greater cellular uptake, inhibited tumor proliferation, and promoted apoptosis in tumor cells better than free curcumin. The results from the intratumoral administration in vivo study demonstrated that an increased dose of GAMCLCL corresponded with decreasing tumor volume and tumor weight. Although GAMCLCL could be administered through an intravenous injection because it displayed good hemocompatibility and low vascular irritation, intratumoral administration is more effective. The reduction in tumor volume and tumor weight by GAMCLCL mentioned earlier was similar to that caused by Adriamycin (a chemotherapy drug) in mice in the positive control group [101]. Clinical trials show that Adriamycin is responsible for multiple side effects, such as frank alopecia, and cessation of beard growth occurred in about 90% of cases, as well as stomatitis, bone marrow depression, alopecia, nausea, vomiting, diarrhea, and fever [102]. Therefore, compared to chemotherapy drugs such as Adriamycin, biodegradable NPs are a much safer alternative exhibiting similar tumor suppression effects.

## Figures and Tables

**Table 1:** Summary Table discussing mechanisms of HIF-1α inhibition, in vitro, in vivo, and clinical evidence, and breast cancer subtype/model used for each phytochemical discussed.

Phytochemical	Mechanism of HIF-1α Inhibition	In Vitro Evidence	In Vivo Evidence	Clinical Evidence	Breast Cancer Subtype/Model
Resveratrol/HS-1793 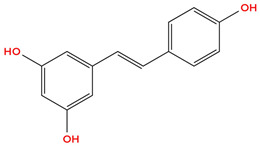	Reduces HIF-1α protein accumulation; inhibits PI3K/Akt, MAPK/ERK [42,43,44,45]	MCF-7, MDA-MB-231: inhibits hypoxia-induced HIF-1α expression [50]	HS-1793 suppressed xenograft growth, downregulated Ki-67 & VEGF [50]	No trials measuring HIF-1α directly; limited bioavailability [51]	MCF-7 (ER+), MDA-MB-231 (TNBC)
EGCG 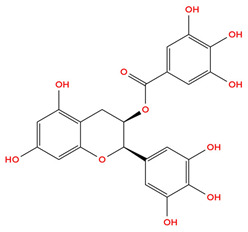	Reduces ROS, promotes HIF-1α degradation via VHL pathway [52,53,54,55,56]	MCF-7: decreased HIF-1α & VEGF, inhibited angiogenesis and cell growth [57]	E0771 (luminal B–like): reduced tumor growth, VEGF, HIF-1α, NFκB [52]	Pre-surgical trial: postmenopausal women with DCIS or early-stage (I/II) invasive breast cancer, reduced Ki-67, no significant changes in apoptosis/angiogenesis [58]	MCF-7 (ER+), E0771 (luminal B–like murine)
Quercetin 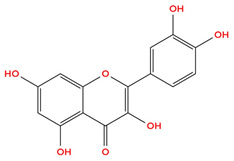	Inhibits HIF-1α protein stabilization through reducing ROS, thereby promoting HIF-1 α hydroxylation [59,60,61,62]	Breast cancer cell lines MCF-7 and SUM159: reduced HIF-1α & VEGF [63]	In vivo: hypoxia-dependent HIF-1α suppression; normal cells unaffected [64]	None; clinical studies lacking [62]	MCF-7 (ER+), SUM159 (TNBC)
Curcumin/EF24 analogue 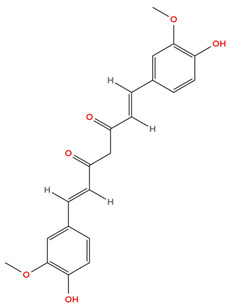	Reduces HIF-1α protein, thereby suppressing HIF-1α effects [65,66]	MDA-MB-231: decrease HIF-1α protein and disrupt hypoxia signaling [66]	Other cancers: HIF-1α downregulation; limited breast cancer results in vivo [65]	Reduced radiation dermatitis in patients with noninflammatory invasive disease or carcinoma in situ; subtype not stratified [67]	MDA-MB-231 (TNBC)
Sulforaphane 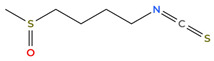	Reduces HIF-1α and VEGF, potentially through the Nrf2/Keap1 pathway; inhibits mammosphere formation [68,69,70]	SUM159 (TNBC) & MCF7 (ER+): decreases mammosphere size/number, HIF-1α, VEGF [70]	SUM159 & MCF7 xenografts: ~50% tumor size reduction, downregulates VEGF/HIF-1α [70]	Postmenopausal patients, early-stage (I/II), predominantly ER+/HER2−: decreases Ki-67, no biomarkers statistically significant [71]	SUM159 (TNBC), MCF7 (ER+), early-stage ER+/HER2− in humans
Berberine 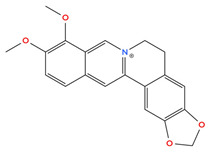	Reduces HIF-1α at mRNA and protein levels; [72,73]	MCF-7: reduces chemoresistance, at >20 μM [73]	Hypoxia-induced, drug-resistant MCF-7 xenografts: decreased tumor volume/weight, AMPK-HIF-1α pathway inhibition [73]	None reported	MCF-7 (ER+)

## Data Availability

No new data were created or analyzed in this study.
